# Effects of Tongue-Strengthening Exercise on Tongue Strength Reserve and Detraining Effects among Healthy Adults: A Randomized Controlled Trial

**DOI:** 10.3390/ijerph19116878

**Published:** 2022-06-04

**Authors:** Hui-Ling Hsiao, Jiunn-Horng Lou, Chun-Chieh Wang, Yun-Ju Lai, Shang-Jung Wu, Yueh-Juen Hwu

**Affiliations:** 1 Department of Nursing, Central Taiwan University of Science and Technology, Taichung 40601, Taiwan; hlhsiao@ctust.edu.tw; 2 Department of Nursing, Hsin Sheng Junior College of Medical Care and Management, Taoyuan 32544, Taiwan; ntp973@hsc.edu.tw; 3 Department of Internal Medicine, Taichung Veterans General Hospital Puli Branch, Nantou 54552, Taiwan; proteinmad@yahoo.com.tw (C.-C.W.); lailai841081@yahoo.com.tw (Y.-J.L.); 4 Department of Eldercare, Central Taiwan University of Science and Technology, Taichung 40601, Taiwan; 5 School of Medicine, National Yang-Ming University, Taipei 11221, Taiwan; 6 Department of Exercise Health Science, National Taiwan University of Sport, Taichung 40404, Taiwan; 7 Department of Nursing, Taichung Veterans General Hospital Puli Branch, Nantou 54552, Taiwan; 8 Department of Healthcare Administration, Central Taiwan University of Science and Technology, Taichung 40601, Taiwan; 9 College of Nursing, Central Taiwan University of Science and Technology, Taichung 40601, Taiwan

**Keywords:** tongue-strengthening exercise, swallowing pressure, tongue strength, presbyphagia

## Abstract

Introduction: Tongue strength reserve is the difference between the maximum isometric pressure (MIP) and swallowing pressure of the tongue. People with decreased tongue strength reserve may have a higher risk of presbyphagia or dysphagia. Thus, this study explored the effects of tongue strengthening exercise (TSE) on tongue strength reserve and detraining effects in healthy adults. Materials and Methods: In total, 102 healthy volunteers without any reported history of speech or swallowing deficits were recruited and assigned to experimental (*n* = 50) and control groups (*n* = 52). Exercises in the experimental group consisted of compressing an air-filled bulb between the tongue and hard palate for 30 min a day, 5 days a week, for 8 weeks. Thereafter, the experimental group underwent a 4-week detraining period. Results: Following the TSE training, posterior tongue strength reserve (F = 4.92, *p* = 0.029) of the experimental group was significantly higher than that of the control group. No significant detraining effects were observed on the MIP and swallowing pressure from 4 weeks after the completion of TSE training. Conclusions: According to the study results, TSE may be an effective approach for improving swallowing function.

## 1. Introduction

The human tongue has two main types of muscle fibers. Type I slow-twitch muscle fibers, located in the posterior tongue, contract slowly, allowing for sustained muscle tone. Type II fast-twitch muscle fibers, located in the anterior tongue, create short, quick bursts of strength [1,2]. Namely, the posterior tongue is responsible for long-lasting tonic activities such as maintaining retroglossal airway patency, while the anterior tongue is responsible for short-lasting bursts of action, such as chewing and speaking [3].

The tongue has a major role in swallowing, as it functions as the primary source of propulsive forces transporting food boluses from the oral cavity into the pharynx [4]. In both the oral and the pharyngeal phases of swallowing, pressing the tongue against the palate is vital. Tongue–palate pressure during maximum isometric tasks is the maximum isometric pressure (MIP) of the tongue or tongue strength. Presbyphagia refers to changes in the swallowing mechanism with age, especially healthy aging. Age-related decline in tongue strength both of anterior and posterior tongue are clearly observed through MIP measures, particularly for individuals aged ≥70 years [5,6,7]. However, healthy older adults do not exhibit reduced tongue–palate pressures during saliva and water swallowing [8,9,10]. The mechanism may be related to the main types of muscle fibers in the anterior and posterior tongue. Most slow-twitch fibers, which are more fatigue resistant and maintain static swallowing pressures, are located in the posterior tongue. Furthermore, saliva or water swallowing are used the tongue pressure amplitudes in the submaximal efforts.

The decline in tongue strength is probably associated with sarcopenia or loss of muscle mass and strength. The muscle mass loss is attributable to atrophy of fast-twitch fibers that provide strength and power. Thus, tongue-to-palate resistance training may improve tongue strength and prevent or halt presbyphagia [11].

Tongue strength reserve represents the difference between the MIP and the swallowing pressure of the tongue. The swallowing pressure appears to be preserved in healthy adults, even when the MIP is reduced [12]. Tongue strength reserve is thus directly related to the MIP. Steele [12] suggested using the difference between the MIP and regular effort saliva swallowing pressure to define tongue strength reserve.

People with decreased tongue strength reserve are more likely to develop presbyphagia or dysphagia resulting from direct or indirect insults to the swallowing system. Declining maximal tongue strength combined with the static demands of swallowing may raise the risk of swallowing difficulties in older adults. This increased tongue weakness is largely due to the existing limited “reserve” as maximal tongue strength grows closer to the demands placed on the system by swallowing.

A systematic review by McKenna et al. conducted to explore the effects of isometric tongue strength training programs on maximal tongue pressures and tongue–palate pressures during swallowing [13]. The results showed that this training can improve maximal tongue pressures in healthy adults and individuals with dysphagia. Data supporting the use of isometric tongue strength training for changing tongue–palate pressures during swallowing are limited [13]. One study also indicated a clear relationship between tongue strengthening exercise (TSE) and MIP [11]. The greater tongue strength will lead to improved swallowing function and may halt or prevent presbyphagia [12]. Since the anterior and posterior tongue have different muscle fibers, most training schemes combine both locations by presuming that different locations activate different muscle groups [14]. TSE-induced increases in the MIP can be reasonably presumed to reflect improved swallowing function.

Studies have shown that TSE increased tongue pressure in healthy participants and this increase was maintained without any further exercise in the subsequent 4-week detraining period [14,15,16,17]. One study examining the effects of directional exercise training on tongue strength [18] reported detraining effects observed from 2 to 4 weeks after the completion of TSE training.

Therefore, we aimed to apply a TSE training program in healthy adults and to investigate the effects of training and detraining on MIP, saliva and water swallowing pressure, and tongue strength reserve in the anterior and posterior tongue.

## 2. Materials and Methods

### 2.1. Study Design and Participants

The present study utilized a two-group pre- and post-test research design. Participants were recruited from a science and technology university in Central Taiwan by using posters and leaflets. The participant inclusion criteria were: age  ≥ 20 years, no history of speech impairments or dysphagia, and no history of medication use for impacting on swallowing or neurological function. In total, 102 healthy volunteers (66 women and 36 men) participated in this study between August 2019 and April 2020. A statistician of the research team randomly assigned each participant to the experimental or control group with a 1:1 allocation by means of a random number generator. From baseline to four weeks of detraining, the participants were blinded to treatment allocation.

This study was approved by the Research Committee Board of Jen-Ai Hospital (Confirmation Number: 107-47), which is guided by local policy, national laws, and the World Medical Association Declaration of Helsinki. This study strictly followed the Consolidated Standards of Reporting Trials (CONSORT) statement for randomized controlled trial, as shown in Table A1. There were no changes to the methods or trial outcomes after trial commencement. Informed consent forms were obtained from all participants prior to their participation in data collection. To avoid bias, no interim analyzes were performed. Since the experimental interventions presented minimal risks, no data monitoring committee would be established, nor would a stopping procedure be implemented. No harm was expected with respect to either treatment.

### 2.2. Training Protocol

The goal of TSE training was to address changes in the MIP, tongue pressure during saliva or water swallowing, and tongue strength reserve. A trained registered nurse provided all instructions to ensure accurate completion of tasks by the participants. During the provision of instructions, each participant was given a demonstration of the task by the same registered nurse, followed by a return demonstration by the participant. The registered nurse also encouraged the participants to complete the task assigned.

The experimental group received an eight-week training program involving TSE, as described by Lin et al. [11], at home. Thereafter, the participants underwent a four-week detraining process. The TSE training program consisted of compressing an air-filled tongue bulb made by the Iowa Oral Performance Instrument (IOPI; Medical LLC, Carnation, WA, USA) between the tongue and hard palate for thirty minutes (a session) a day, five days a week, for eight weeks. Each session included 30 training repetitions each for both locations of the tongue. The participants exercised the anterior and posterior regions of the tongue one after the other.

The tongue is a muscular hydrostat and generally fatigue resistant [13]. In TSE training, greater resistive loads produce greater strength gains [1]. To perform TSE, the participants were asked to compress the air-filled tongue bulb as hard as possible for ten seconds. Ten seconds is viewed as the bottom line for maintaining adequate tongue function [5]. During TSE training, the same bulb with permanent markers was utilized to keep the exact anterior and posterior tongue locations. The examiners tracked training records of participants each week. All participants reported 100% compliance.

### 2.3. Detraining

After the eight-week TSE training, the participants in the experimental group were informed to continue their normal lifestyles and avoid any type of TSEs for four weeks. Since one of the study aims was to investigate the detraining effects after a training program, we analyzed the effects only in the experimental group.

### 2.4. Measurement

Measurements of tongue pressure were administrated by the IOPI (IOPI Medical, Redmond, WA, USA), calibrated as per the manufacturer’s instructions before study initiation and on a monthly basis throughout data collection. The device was also recalibrated whenever it was noticed to have a resting pressure above zero.

The tongue bulb was placed directly behind the central incisors to measure the anterior tongue pressure, and was placed in line with the first molars while measuring the posterior tongue pressure [19]. MIPs and swallowing pressures of the tongue were measured at baseline and weeks 2, 4, 6, and 8. The experimental group received an additional two measurements at 2 and 4 weeks of detraining. The measures order was consistent across all evaluation sessions. MIPs of the anterior and posterior tongue were obtained first, followed by saliva swallowing pressure and water (5 mL) swallowing pressure. A five-minute rest period was maintained between measurements to avoid fatigue. The tongue strength was measured three times in succession, and the maximum value was adopted as the tongue strength (Table 1). Two external examiners completed the outcome evaluation.

The same two examiners performed all measurements using the IOPI on five occasions. The two measurements of detraining effects were conducted by one of the aforementioned examiners. The conditions of each evaluation remained the same for all tests. To prevent muscle injury and muscle soreness after the evaluation, all participants attended two familiarization trials before study initiation.

### 2.5. Validity and Reliability

The study design ensured that the findings were valid and reliable. Firstly, the research team carefully discussed and developed the study protocol to meet the standards. Three experts in neurology, anatomy/physiology, and gerontological nursing verified the content validity of the training protocol. Secondly, the same researcher administered the intervention to all participants in the experimental group to maintain consistency. Compliance with the protocol was ensured by monitoring the home practice records through a research meeting conducted after the test. Thirdly, outcome examiners were trained to follow and comply with the instructions of IOPI user manual. Finally, a statistician determined the data accuracy, monitored the reports, and contributed to the analysis.

### 2.6. Statistical Analysis

Chi-square test and independent *t*-tests were utilized for testing the homogeneity of two group baselines. Training effects on tongue strength were analyzed using generalized linear modeling (GLM) and a repeated-measures analysis of covariance (RANCOVA). Since a RANCOVA model could include all considered variables, there was the overall confidence interval of 0.95 and the overall significance level of 0.05.

A repeated-measures analysis of variance (ANOVA) with the least significant difference was used to analyze the detraining effects. The data were analyzed using the software program IBM SPSS Statistics (Armonk, NY, USA).

The sample size was estimated utilizing the software G*Power 3 (Dusseldorf, Germany) for repeated-measures ANOVA regarding two groups, five repeated measurements, a medium effect size of 0.3, power of 0.95, and type I error rate of 0.05. The required total sample size was 90.

## 3. Results

**Figure 1:**
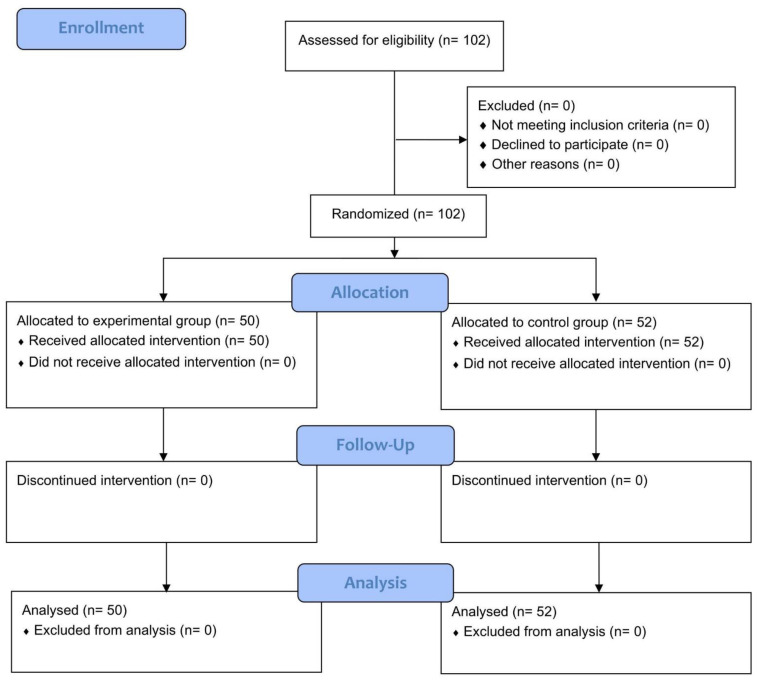
Flow diagram of the study design.

Considering the dropout rate of 10% for the experimental group and 14% for the control group [20], we recruited a total of 102 healthy adults for eligibility and assigned 50 and 52 participants to the experimental and control groups, respectively. No participant withdrew from the program, and the experimental group completed the eight-week TSE training program and four-week follow-up (Figure 1).

### 3.1. Baseline Characteristics

The mean reported age of the 102 participants was 37.24 ± 14.74 years (range: 20–59). The participants were predominantly women (64.7%). Their average body height, body weight, and body mass index were 163.36 ± 8.27 cm, 64.76 ± 13.54 kg, and 24.12 ± 4.01 kg/m^2^, respectively. The average MIPs of the anterior and posterior tongue were 56.41 ± 14.17 kPa and 52.76 ± 13.09 kPa, respectively, and their corresponding average saliva swallowing pressures were 47.74 ± 15.91 kPa and 47.27 ± 15.25 kPa, respectively. The average water swallowing pressures of the anterior and posterior tongue were 43.22 ± 16.90 kPa and 41.07 ± 15.89 kPa, respectively, and their corresponding values of the average tongue strength reserve were 8.68 ± 2.10 kPa and 5.49 ± 1.77 kPa, respectively (Table 2).

No statistically significant differences in the demographics were observed between the two groups, indicating that the groups were homogeneous. The two pretest values of the tongue strength were larger for the experimental group than for the control group. No significant differences were observed in the two pretest values of saliva swallowing pressures for both tongue locations between the two groups. By contrast, for the water swallowing pressures of both locations of the tongue, the two pretest values of water swallowing pressures for both locations were larger in the control group than in the experimental group. No significant differences were observed in the two pretest values of the tongue strength reserve between the two groups (Table 2).

### 3.2. Training Effects

The MIP, saliva and water swallowing pressures, and tongue strength reserved for both tongue locations were compared among the five points in time (at the baseline and weeks 2, 4, 6, and 8 of training) to determine the TSE training effects over time. We used GLM and RANCOVA to explore the group effects on the outcome variables. In RANCOVA, these four posttest values were dependent variables, whereas the pretest values were covariate, and the group variable was the independent variable.

#### 3.2.1. MIP of Tongue

After the eight-week training period, differences in MIPs of the anterior tongue (F = 4.87, *p* = 0.030) and posterior tongue (F = 4.67, *p* = 0.033) between the groups confirmed the significant group effects. The postintervention mean value of MIP was higher in the experimental group than in the control group. In terms of time effect, RANCOVA revealed a statistically significant improvement in MIPs of the anterior tongue (F = 3.41, *p* = 0.016) and posterior tongue (F = 18.56, *p* < 0.001), respectively. Post hoc tests verified that the MIPs of both tongue locations at 2 weeks were significantly greater than those at the baseline. No significant interaction (group × time) effects were noted on MIPs of both tongue regions (Table 3).

#### 3.2.2. Swallowing Pressure of Tongue

RANCOVA did not reveal a significant main effect of group, main effect of time, or interaction (Group × time) effect on the saliva swallowing pressure. However, significant main effects of the group were noted on the water swallowing pressure of the anterior tongue (F = 8.06, *p* = 0.005) and posterior tongue (F = 7.32, *p* = 0.008), respectively. After eliminating the interference of group differences at the pretest, the average water swallowing pressures of the anterior tongue at 2, 4, 6, and 8 weeks were 46.10, 45.18, 46.60, and 46.84 kPa for the control group, respectively, and 41.51, 41.00, 43.36, and 44.92 kPa for the experimental group, respectively, indicating that these water swallowing pressures were higher in the control group than in the experimental group at all time points. The average water swallowing pressures of the posterior tongue at 2, 4, 6, and 8 weeks were 44.78, 44.07, 46.34, and 46.83 kPa for the control group, respectively, and 41.33, 45.06, 45.68, and 43.51 kPa for the experimental group, respectively, indicating that these water swallowing pressures were higher in the control group than in the experimental group.

In terms of the time effect, significant improvements were noted in the water swallowing pressure of the posterior tongue (F = 4.63, *p* = 0.003). Post hoc tests verified that the water swallowing pressures of the posterior tongue at 2 weeks were significantly greater than those at the baseline. The RACOVA results indicated no significant interaction (Group × Time) effects on the water swallowing pressure of both locations of the tongue in the two groups (Table 3).

#### 3.2.3. Tongue Strength Reserve

**Figure 2:**
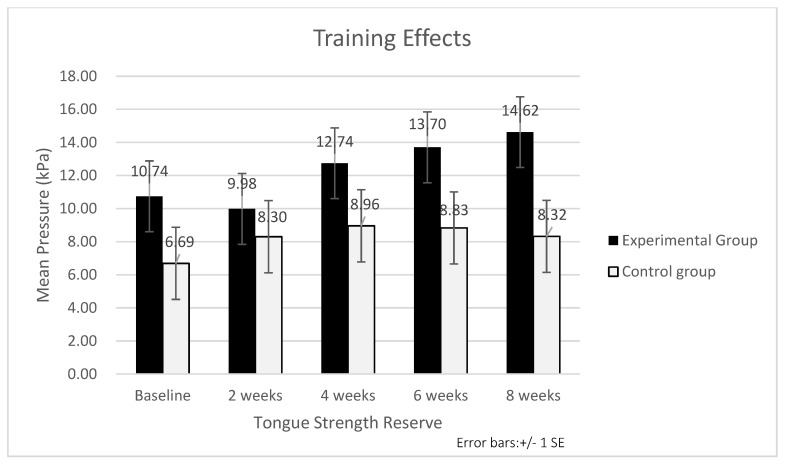
Tongue strength reserve measures changed following 8 weeks of training for two groups.

The RANCOVA results indicated a significant main effect of the groups on the tongue strength reserve of the posterior tongue (F = 4.92, *p* = 0.029), with the postintervention mean value being higher in the experimental group than in the control group (Table 3 and Figure 2). The change in the tongue strength reserve of the posterior tongue over time was significant in both groups (F = 6.26, *p* < 0.001). Post hoc tests verified that the tongue strength reserve of the posterior tongue was significantly increased at the 4th week compared with the baseline. No significant interaction (group × time) effects were noted on the tongue strength reserve of the posterior tongue in the two groups. RANCOVA did not reveal a significant main effect of the group, main effect of the time, or interaction (Group × Time) effect on the tongue strength reserve of the anterior tongue (Table 3).

**Figure 3:**
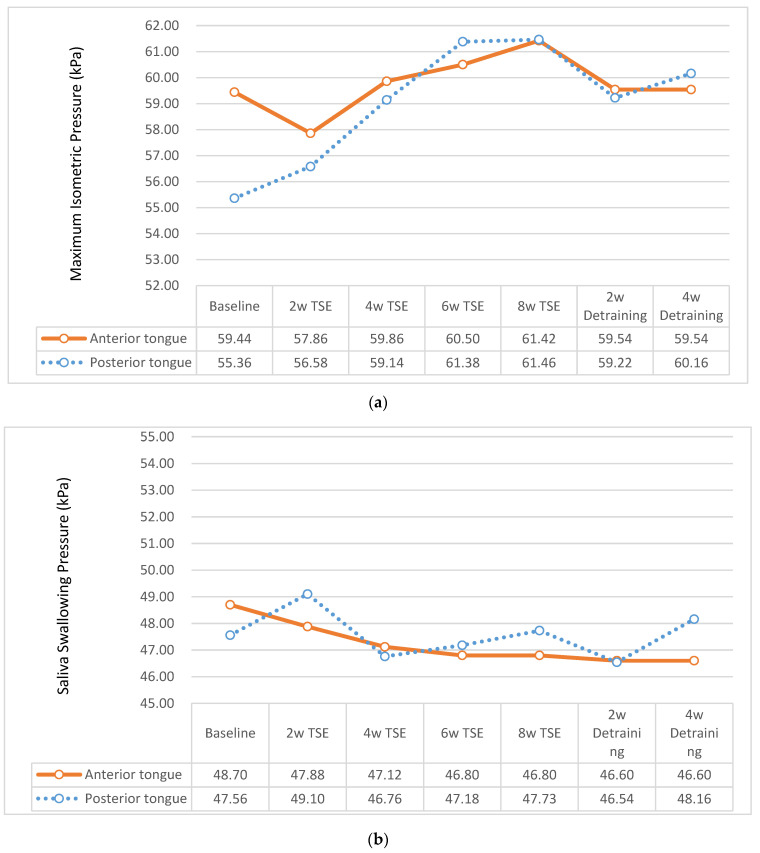

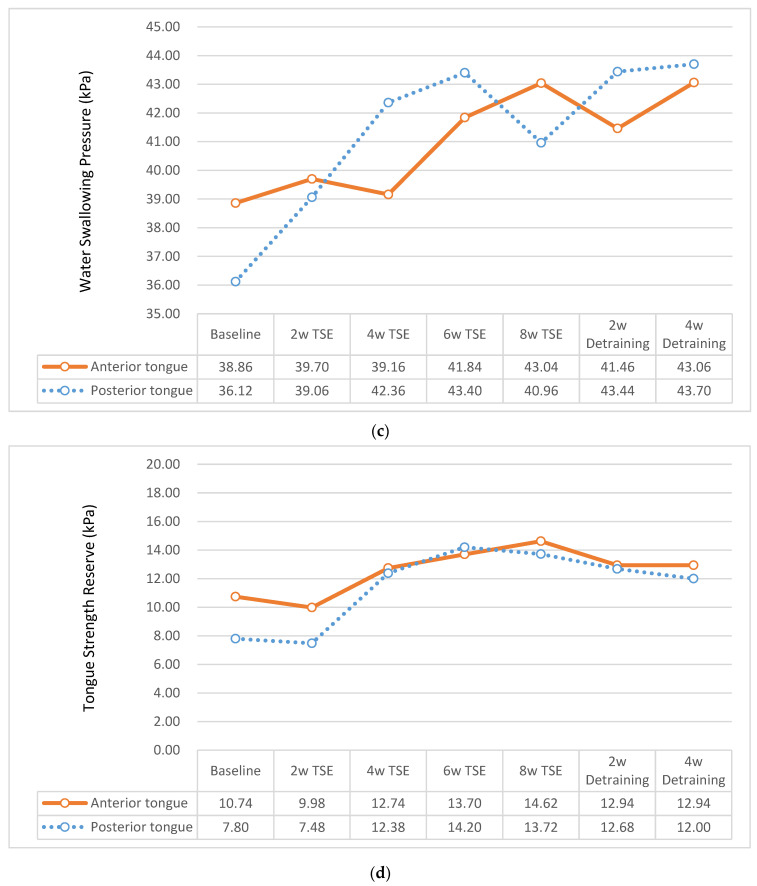
Detraining effects of the tongue strength by location of the tongue: (**a**) maximum isometric pressure (MIP), (**b**) saliva swallowing pressure, (**c**) water (5 mL) swallowing pressure, and (**d**) tongue strength reserve. 2w: 2 weeks; 4w: 4 weeks; 6w: 6 weeks; 8w: 8 weeks; TSE: tongue-strengthening exercises.

After a four-week detraining period, no significant decreases in the MIP, saliva and water swallowing pressures, and tongue strength reserve were noted for both tongue locations compared with the eight-week training (Table 4). When detraining for four weeks, the tongue strength is expressed as a percent of the tongue strength after the eight-week training period, and the MIP decreased by 3% for the anterior tongue and 2% for the posterior tongue (Figure 3a). The saliva swallowing pressure decreased by 0.4% for the anterior tongue and increased by 0.9% for the posterior tongue (Figure 3b). The water swallowing pressure increased by 0.04% for the anterior tongue and by 7% for the posterior tongue (Figure 3c). The tongue strength reserve decreased by 11% for the anterior tongue and by 12% for the posterior tongue (Figure 3d).

## 4. Discussion

The present study explored the changes in the MIP, saliva and water swallowing pressures, and tongue strength reserve for both anterior tongue and posterior tongue among healthy adults with regards to 8-week TSE training and then 4-week detraining.

### 4.1. Effects of TSE on Tongue Strength Reserve

The present study revealed that, following eight weeks of TSE training, the participants in the experimental group demonstrated substantial gains in the MIP of the tongue, with no change in saliva swallowing pressure for both locations of tongue and water swallowing pressure of the anterior tongue, and had higher tongue strength reserve in the posterior tongue than the control group, which were congruent with previous studies [8,10,21,22]. According to the previous literature [8,10,23,24,25], the MIP of the tongue decreases with changes in healthy aging, which includes the anatomy and physiological mechanisms with regards to head and neck, whereas swallowing pressure of tongue does not, resulting in a reduction in tongue strength reserve.

Tongue strength reserve is a term used to describe the difference in pressure between the MIP and the pressure generated in the swallowing tasks. Robbins et al. were the first to present that swallowing pressures appear to be preserved in normal aging, even when the MIPs were decreased [26]. Reductions in tongue strength reserve, due to decreases in MIPs, will increase the risk of developing functional dysphagia [26]. Our findings indicate that TSE training can improve the MIP of the tongue and the tongue strength reserve of the posterior tongue but has no effect either on saliva swallowing pressure or on water swallowing pressure of the anterior tongue. Therefore, TSE training can compensate for age-related changes in swallowing function and prevent or delay the decline of tongue strength reserve for safe swallowing.

Steele mentioned that, in addition to MIP measurement, the tongue strength reserve should also be assessed when evaluating tongue functions, because reduced tongue strength reserve is thought to pose a risk for developing dysphagia [27]. As saliva swallows involve less risk of aspiration than water swallows, it is recommended that tongue strength reserve measurements should use a short series of MIPs and saliva swallows [27]. The current study also confirmed that, after eight weeks of TSE training, the saliva swallowing pressure remained stable without statistically significant changes. These results supported that it is feasible to evaluate tongue strength reserve using the measures of saliva swallowing pressure.

### 4.2. Effects of TSE on Tongue Swallowing Pressure

Although the participants did not exercise their tongue pressure during swallowing, their water swallowing pressures of posterior tongue illustrated a significant improvement following the TSE training. The reasons listed as follows might explain changes in water swallowing pressures in the posterior tongue that appeared during the two weeks of intervention for both groups. Since the tongue is a muscular hydrostat organ, when participants pushed as hard as they could against the bulb, muscles essential to swallowing initiation are recruited, resulting in a more vigorous swallow [28]. In addition, the measurement of the anterior and posterior tongue strength under the context of maximum isometric pressure, and the swallowing pressure of saliva and water can cause the adaptation of other muscle fibers to keep the volume of this organ constant during movement and exercising [14]. Subsequently, period measurements can lead to possible training of the tongue muscle. Hence, the increased water swallowing pressure may associate with the improvement in tongue muscular strength through the TSE training [17].

Given that the data collection protocol involved measuring MIP, saliva swallowing pressure, and water swallowing pressure, the possibility of order effects cannot be excluded. Participants might feel the need to exert more strength to complete the task. Thus, the water swallowing pressure of the posterior tongue increased at the end of training.

The result showed that the control group had a significantly higher mean water swallowing pressure than the experimental group. Therefore, the control group generally swallowed water with more tongue force than the experimental group. The reason may be that, after eight weeks of TSE training in the experimental group, the maximum tongue strength increased, and the submaximal pressure required for water swallowing decreased.

Youmans and colleagues found the values of swallowing pressures of various liquids from thin to pureed consistency were 50–60% of the maximum tongue strength [10]. However, the participants used 76–89% of the maximum tongue strength for swallowing in this study. It might be possible that participants found an air-filled ball placed in the mouth during swallowing unnatural, which influenced the pressure amplitudes. Additionally, while participants were asked to swallow saliva and water as naturally as possible, we cannot exclude the possibility that prior directions to carry out isometric tasks with maximum effort affected the effort used during the water swallowing tasks.

The anterior human tongue is predominantly type II muscle fibers, and the posterior human tongue is predominantly Type I muscle fibers [21]. Type I slow-twitch muscle fibers are well-suited for closing off the upper airway during mastication to prevent premature advancement of the bolus. Type II fast-twitch muscle fibers are responsible for bolus manipulation and propulsion during swallowing [3].

Studies on limb muscle training show that the strengthening effects diminish over time during the detraining period [17]. The tongue is made up completely of muscle and works independently of the skeleton, which is different from the limb muscles [17,29]. Instead, movement is accomplished through a complex pattern of contractions of fibers aligned in intersecting planes [30]. The underlying mechanisms may explain the differences in detraining effects between tongue and limb muscles.

The studies relevant to studying the detraining effects of TSE were Clark et al. [18], Oh [17], and Van den Steen et al. [14]. The comparison of TSE training and detraining effects among the different studies is shown in Table 5. The former study investigated the specific effects of three different types of tongue exercises (elevation, protrusion, and lateralization) utilizing a tongue blade to assess the tongue strength among healthy adults The results showed that the tongue strength significantly improved following the 9-week training. The participants demonstrated a significant decrease in tongue strength from 23.2 days (range = 18–31 days) after the completion of training. The tongue strength remained numerically higher than that of pretraining but not significantly different [18]. Oh recruited ten young healthy volunteers (21–35 years) [17]. Following the completion of eight weeks of TSE, a 28-week detraining period was continued. After a 28-week detraining period, the participants had significantly lower mean values for all tongue variables than at eight weeks of training but significantly higher than at the baseline. In the study conducted by Van den Steen et al. exploring the effects of two tongue regions with TSE training, 16 healthy elderly volunteers (70–95 years) completed eight weeks of TSE, and the detraining was measured four weeks after the completion of TSE training [14]. The results showed there were no significant detraining effects observed at 4 weeks after the completion of the TSE training [14] (Table 5). In the current study, 50 healthy adults (20–59 years) completed an eight-week TSE, and there were no significant detraining effects four weeks after the last TSE session. The discrepancies between the four studies were exercise selection, duration, frequency, and training intensity or volume. The strengthening effects attained at eight weeks began to decrease significantly after eight weeks of detraining. The retraining exercise regimen is thus suggested after eight weeks of the detraining period.

### 4.3. Limitations

This study has several limitations that can be considered as future research suggestions. Firstly, the ceiling effect was observed because most of the participants were young; therefore, the scores of tongue strength clustered toward the high end of the measurements. Hence, the variance of intervention was not estimated above a certain level [31]. Secondly, the study participants were healthy adults without swallowing difficulties recruited from a science and technology university in Central Taiwan. They may not reflect the characteristics of the entire population, and the findings should be interpreted with caution, since the results may not apply to other groups with medical conditions. Thirdly, the proportion of male to female participants in the present study was 36:66, and this may confound sex differences in response to training. Finally, this study addressed the changes in tongue strength among healthy adults following TSE training. The findings do not speak to whether the performance of healthy adults is typical of those with orofacial weakness. Given these facts, the benefits of tongue strengthening exercises for building a tongue strength reserve and restoring more normal swallowing functions are still to be clearly demonstrated.

### 4.4. Suggestions

Participant adherence was supervised only by self-reporting. Training fidelity of the participants may be ensured through supervision. Examining the differential effects of supervised versus unsupervised exercise is suggested for future studies on TSE. Detraining effects after TSE training on the tongue strength among older adults or patients with swallowing impairments need to be explored. The follow-up period must be prolonged by more than four weeks to evaluate the detraining effects. Periodic exercise or retraining is required after prolonged TSE to improve or maintain the exercise effect. Future studies should comprise equal numbers of males and females so that training-specific sex differences may be assessed for tongue strength reserves.

## 5. Conclusions

In summary, the results of this randomized controlled study indicated an improvement in MIPs of the tongue strength after eight weeks of TSE training in healthy adults. There was no significant difference in tongue pressure during swallowing, except for water swallowing of posterior tongues. Our data confirmed that TSE makes it possible to increase the tongue strength reserve in the posterior tongue. Furthermore, we found no detraining effects on the MIPs, saliva and water swallowing pressures, and tongue strength reserve. Therefore, TSE can be an effective approach to prevent or halt the progression of presbyphagia or dysphagia and reduce healthcare expenditure.

## Figures and Tables

**Table 1:** Tasks, categories, and repetitions.

Task	Bulb-Position	Repetitions
Isometric	Anterior	3 presses
	Posterior	3 presses
<5 min break>		
Saliva Swallowing	Anterior	3 presses
	Posterior	3 presses
<5 min break>		
Water (5 mL) Swallowing	Anterior	3 presses
	Posterior	3 presses

**Table 2:** Demographics and outcome variables of participants at the baseline (*n* = 102).

Variable	Total (*N* = 102)	Experimental Group (*n* = 50)	Control Group (*n* = 52)	t/χ^2^	*p*
	*N* (%)/Mean ± SD	*n* (%)/Mean ± SD	*n* (%)/Mean ± SD		
**Gender**				0.31	0.575
Male	36 (35.3)	19(38.0)	17(32.7)		
Female	66 (64.7)	31(62.0)	35(67.3)		
**Age** (years)	37.24 ± 14.74	37.68 ± 16.25	36.81 ± 13.28	0.30	0.767
**Body Height** (cm)	163.36 ± 8.27	163.41 ± 8.84	163.32 ± 7.75	0.05	0.957
**Body Weight** (kg)	64.76 ± 13.54	64.92 ± 14.83	64.60 ± 12.32	0.12	0.906
**BMI**	24.12 ± 4.01	24.14 ± 4.37	24.10 ± 3.68	0.05	0.960
**MaximumIsometric Pressure** (MIP, kPa)					
Anterior tongue	56.41 ± 14.17	59.44 ± 11.97	53.50 ± 15.57	2.15	0.034
Posterior tongue	52.76 ±13.09	55.36 ± 13.53	50.27 ± 12.28	1.99	0.049
**Saliva Swallowing Pressure** (kPa)					
Anterior tongue	47.74 ± 15.91	48.70 ± 17.71	46.81 ± 14.07	0.60	0.551
Posterior tongue	47.27 ± 15.25	47.56 ± 16.56	47.00 ± 14.03	0.19	0.854
**Water Swallowing Pressure** (kpa)					
Anterior tongue	43.22 ± 16.90	38.86 ± 17.07	47.42 ± 15.79	−2.63	0.010
Posterior tongue	41.07 ± 15.89	36.12 ± 16.74	45.83 ± 13.55	−3.22	0.002
**Tongue Strength Reserve** (kPa)					
Anterior tongue	8.68 ± 2.10	10.74 ± 2.06	6.69 ± 2.14	1.36	0.177
Posterior tongue	5.49 ± 1.77	7.80 ± 1.92	3.27 ± 1.63	1.80	0.074

Note: Continuous variables are expressed as the mean ± SD. Independent *t*-tests were used to compare the variables between groups. The categorical variables are presented by frequency (percent), and their homogeneity was tested using χ^2^ tests.

**Table 3:** Training effects of tongue-strengthening exercise.

Variables	Sphericity Test (*p*)	Mean Square	Degree of Freedom	F	*p*	LSD Test ^b^
**Maximum Isometric** **Pressure** (MIP, kPa)						
*Anterior tongue*	<0.001					
Group		2774.81	1	4.87	0.030	
Time (1, 2, 3, 4, 5) ^a^		209.30	3.16	3.41	0.016	5 > 3 > 2 > 1
Group × Time		23.49	3.16	0.38	0.776	
*Posterior tongue*	<0.001					
Group		2093.85	1	4.67	0.033	
Time (1, 2, 3, 4, 5)		1018.65	3.19	18.56	<0.001	5 > 3 > 2 > 1
Group × Time		24.56	3.19	0.45	0.731	
**Saliva Swallowing** **Pressure** (kPa)						
*Anterior tongue*	<0.001					
Group		36.06	1	0.05	0.827	
Time (1, 2, 3, 4, 5)		32.22	3.44	0.31	0.846	
Group × Time		75.75	3.44	0.72	0.558	
*Posterior tongue*	<0.001					
Group		16.53	1	0.02	0.883	
Time (1, 2, 3, 4, 5)		33.43	3.25	0.34	0.814	
Time × Group		112.45	3.25	1.13	0.338	
**Water Swallowing** **Pressure** (kPa)						
*Anterior tongue*	<0.001					
Group		6819.09	1	8.06	0.005	
Time (1, 2, 3, 4, 5)		199.17	3.03	1.42	0.238	
Group × Time		54.02	3.03	0.38	0.767	
*Posterior tongue*	<0.001					
Group		6517.02	1	7.32	0.008	
Time (1, 2, 3, 4, 5)		528.41	3.04	4.63	0.003	4 > 2 > 1, 3 > 1, 5 > 1
Group × Time		166.78	3.04	1.48	0.220	
**Tongue strength reserve**						
*Anterior tongue*	<0.001					
Group		2178.21	1	2.93	0.090	
Time (1, 2, 3, 4, 5)		189.39	3.47	1.46	0.219	
Group × Time		83.93	3.47	0.65	0.607	
*Posterior tongue*	0.002					
Group		2482.45	1	4.92	0.029	
Time (1, 2, 3, 4, 5)		684.41	3.54	6.26	<0.001	3 > 1, 4 > 1, 5 >1, 4 > 2, 5 > 2
Group × Time		149.57	3.54	1.37	0.249	

^a^ 1 = baseline, 2 = training 2 weeks, 3 = training 4 weeks, 4 = training 6 weeks, and 5 = training 8 weeks. ^b^ LSD = least significant differences; 5 > 1 means the mean of the 5th test (training 8 weeks) is significantly greater than that at the baseline under the LSD methodology.

**Table 4:** Detraining effects of the tongue pressure (*n* = 50).

Variable	M ± SE	Mean Square	df	F	*p*	LSD Test
**Maximum Isometric** **Pressure** (MIP, kPa)						
*Anterior tongue*		80.56	4.42	1.60	0.168	
1. baseline	59.44 ± 1.69					
2. Training 2 weeks	57.86 ± 1.94					
3. Training 4 weeks	59.86 ± 1.53					
4. Training 6 weeks	60.50 ± 1.70					
5. Training 8 weeks	61.42 ± 1.58					
6. Detraining 2 weeks	59.54 ± 1.66					
7. Detraining 4 weeks	59.54 ± 1.45					
*Posterior tongue*		393.01	4.10	7.07	<0.001	6 > 1, 6 > 2, 6 > 4, 6 > 5, 7 > 1, 7 > 2
1. baseline	55.36 ± 1.91					
2. Training 2 weeks	56.58 ± 1.79					
3. Training 4 weeks	59.14 ± 1.58					
4. Training 6 weeks	61.38 ± 1.49					
5. Training 8 weeks	61.46 ± 1.42					
6. Detraining 2 weeks	59.22 ± 1.42					
7. Detraining 4 weeks	60.16 ± 1.32					
**Saliva Swallow Pressure** (kPa)						
*Anterior tongue*		31.31	6	0.30	0.937	
1. baseline	48.70 ± 2.50					
2. Training 2 weeks	47.88 ± 2.24					
3. Training 4 weeks	47.12 ± 2.12					
4. Training 6 weeks	46.80 ± 2.34					
5. Training 8 weeks	46.80 ± 2.24					
6. Detraining 2 weeks	46.60 ± 2.31					
7. Detraining 4 weeks	46.60 ± 2.26					
*Posterior tongue*		49.53	4.63	0.39	0.845	
1. baseline	47.56 ± 2.34					
2. Training 2 weeks	49.10 ± 2.17					
3. Training 4 weeks	46.76 ± 2.02					
4. Training 6 weeks	47.18 ± 2.09					
5. Training 8 weeks	47.73 ± 2.12					
6. Detraining 2 weeks	46.54 ± 2.13					
7. Detraining 4 weeks	48.16 ± 1.96					
**Water Swallow Pressure** (kPa)						
*Anterior tongue*		259.92	3.65	1.41	0.236	
1. baseline	38.86 ± 2.41					
2. Training 2 weeks	39.70 ± 2.13					
3. Training 4 weeks	39.16 ± 2.58					
4. Training 6 weeks	41.84 ± 2.45					
5. Training 8 weeks	43.04 ± 2.56					
6. Detraining 2 weeks	41.46 ± 2.57					
7. Detraining 4 weeks	43.06 ± 2.34					
*Posterior tongue*		558.67	4.28	4.03	0.003	6 > 1, 6 > 2, 7 > 1, 7 > 2
1. baseline	36.12 ± 2.36					
2. Training 2 weeks	39.06 ± 2.28					
3. Training 4 weeks	42.36 ± 2.58					
4. Training 6 weeks	43.40 ± 2.42					
5. Training 8 weeks	40.96 ± 2.61					
6. Detraining 2 weeks	43.44 ± 2.23					
7. Detraining 4 weeks	43.70 ± 2.28					
**Tongue strength reserve**						
*Anterior tongue*		162.60	4.87	1.12	0.351	
1. baseline	10.74 ± 2.06					
2. Training 2 weeks	9.98 ± 2.21					
3. Training 4 weeks	12.74 ± 1.98					
4. Training 6 weeks	13.70 ± 2.62					
5. Training 8 weeks	14.62 ± 2.22					
6. Detraining 2 weeks	12.94 ± 2.29					
7. Detraining 4 weeks	12.94 ± 2.21					
*Posterior tongue*		452.66	4.92	3.34	0.006	6 > 1, 6 > 2
1. baseline	7.80 ± 1.92					
2. Training 2 weeks	7.48 ± 2.01					
3. Training 4 weeks	12.38 ± 1.95					
4. Training 6 weeks	14.20 ± 1.97					
5. Training 8 weeks	13.72 ± 1.97					
6. Detraining 2 weeks	12.68 ± 2.11					
7. Detraining 4 weeks	12.00 ± 1.85					

Note: LSD = least significant difference; SE = standard error of the mean. 1 = baseline, 2 = training 2 weeks, 3 = training 4 weeks, 4 = training 6 weeks, 5 = training 8 weeks, 6 = detraining 2 weeks, and 7 = detraining 4 weeks.

**Table 5:** Comparisons of the TSE training effects and detraining effects.

Study/Method/Aim	Participants	Interventions	Outcomes
Clark et al. [18]; Randomization of assignment to the sequential TSE group (*n* = 29) or concurrent TSE group (*n* = 10) Aim: To verify the effects of TSE on tongue strength, and whether the strength gains maintain after exercise discontinued	39 healthy adults; 17 males and 22 females; Mean = 37.8 years, range = 18–67 years	Participants of both groups received three different types of tongue exercise (elevation, protrusion, and/or lateralization) for 9 weeks. Participants performed 30 repetitions of tongue exercise a day, 7 days a week. Each repetition included a contraction for 1 s. 19 participants took part in the detraining process. Participants were measured the MIP and cheek strength weekly by IOPI.	All variables of tongue pressure were improved following the TSE training, but cheek strength did not change with TSE training. Significant detraining effects on tongue strength were observed from an average of 23.2 days after the completion of TSE training.
Oh [17]; pre-experimental research design Aim: To assess the effects of TSE and detraining effects on tongue strength and tongue pressure during effortful swallowing.	10 young healthy volunteers; 3 males and 7 females; Mean = 25.8 years, range = 21–35 years	Participants received 30-min TSE a day, 3 days a week, for 8 weeks. All study participants pressed the tongue against the bulb for 2 s. After finishing the 8-week training, 28-week detraining was followed. MIPs of tongue strength and effort swallowing pressure were measured by IOPI on 12 time points (at baseline and weeks 2, 4, 6, 8 of training, and at weeks 4, 8, 12, 16, 20, 24, 28 of detraining).	TSE increased the MIPs of tongue strength and effortful swallowing pressure. All variables of tongue pressure were significantly decreased after 28 weeks of detraining compared with 8-week training. The significantly decreased gains of strength in anterior tongue appeared at weeks 8 of detraining.
Van den Steen et al. [14]; Assignment to anterior TSE group (*n* = 7) or posterior TSE group (*n* = 9) using convenience sampling Aim: To explore the training effects of anterior and posterior TSE on tongue strength and the detraining effects.	16 older adults in nursing home; 8 males and 8 females; Mean = 84 years, range = 70–95 years	7 participants received anterior TSE and 9 participants posterior TSE. Participants received TSE session 3 times a week, for 8 weeks. Each session included 120 repetitions of tongue pressure exercises. Participants of both groups pressed the tongue against the bulb for 3 s. MIPs were measured by IOPI on 5 time points (at baseline and weeks 4, 8 of training, and at weeks 4 of detraining).	MIPs in anterior and posterior tongue increased significantly in both treatment arms. No significant detraining effects were observed from 4 weeks after the completion of TSE training.
Present study; Randomization of assignment to the experimental group (*n* = 50) or control group (*n* = 52) Aim: To explore the effects of TSE on MIPs of tongue strength, tongue pressure during saliva and water swallowing, tongue pressure reserve and to measure possible detraining effects.	102 healthy adults; 36 males and 66 females; Mean = 37.2 years, range = 20–59 years	Participants were randomly assigned to either the experimental group or the control group. The participants in the experimental group received 30-min TSE session a day, 5 days per week, for 8 weeks. Each TSE session included 30 repetitions for both locations of tongue, respectively. The participants in the experimental group pressed the tongue against the bulb for 10 s. After finishing the 8-week training, 4-week detraining was followed for the experimental group. MIPs of tongue strength and tongue pressures during saliva and water swallowing were measured by IOPI on 5 time points (at baseline and weeks 2, 4, 6, 8 of training) for both group, and 2 time points (at 2, 4 weeks of detraining) for the experimental group.	The experimental group illustrated significant improvements in MIPs of tongue strength. There was no significant difference in the tongue pressure during swallowing except water swallowing in posterior tongue. After 4 weeks detraining, there was no significant decrease in MIPs of tongue strength and tongue pressure during swallowing, compared with 8-week training.

Source: compiled by the authors.

## Data Availability

The datasets used and/or analyzed during the present study are available from the corresponding author upon reasonable request.

## Appendix A

**Table A1:** CONSORT checklist of the study.

Section/Topic	Item No	Checklist Item	Reported on Page No
**Title and abstract**			
	1a	Identification as a randomised trial in the title	1
	1b	Structured summary of trial design, methods, results, and conclusions (for specific guidance see CONSORT for abstracts)	1
**Introduction**			
Background and objectives	2a	Scientific background and explanation of rationale	1–2
	2b	Specific objectives or hypotheses	2
**Methods**			
Trial design	3a	Description of trial design (such as parallel, factorial) including allocation ratio	3–4; Figure 1
	3b	Important changes to methods after trial commencement (such as eligibility criteria), with reasons	3
Participants	4a	Eligibility criteria for participants	3
	4b	Settings and locations where the data were collected	3
Interventions	5	The interventions for each group with sufficient details to allow replication, including how and when they were actually administered	3–4
Outcomes	6a	Completely defined prespecified primary and secondary outcome measures, including how and when they were assessed	3–4
	6b	Any changes to trial outcomes after the trial commenced, with reasons	3
Sample size	7a	How the sample size was determined	4
	7b	When applicable, explanation of any interim analyses and stopping guidelines	NA
Randomisation:			
Sequence generation	8a	Method used to generate the random allocation sequence	3
	8b	Type of randomisation; details of any restriction (such as blocking and block size)	3
Allocation concealment mechanism	9	Mechanism used to implement the random allocation sequence (such as sequentially numbered containers), describing any steps taken to conceal the sequence until interventions were assigned	3
Implementation	10	Who generated the random allocation sequence, who enrolled participants, and who assigned participants to interventions	3
Blinding	11a	If done, who was blinded after assignment to interventions (for example, participants, care providers, and those assessing outcomes) and how	3
	11b	If relevant, description of the similarity of interventions	NA
Statistical methods	12a	Statistical methods used to compare groups for primary and secondary outcomes	4
	12b	Methods for additional analyses, such as subgroup analyses and adjusted analyses	4
**Results**			
Participant flow (a diagram is strongly recommended)	13a	For each group, the numbers of participants who were randomly assigned, received intended treatment, and were analysed for the primary outcome	5; Figure 1
	13b	For each group, losses and exclusions after randomisation, together with reasons	5; Figure 1
Recruitment	14a	Dates defining the periods of recruitment and follow-up	3
	14b	Why the trial ended or was stopped	3
Baseline data	15	A table showing baseline demographic and clinical characteristics for each group	6–7
Numbers analysed	16	For each group, number of participants (denominator) included in each analysis and whether the analysis was by original assigned groups	3, 5–6; Figure 1
Outcomes and estimation	17a	For each primary and secondary outcome, results for each group, and the estimated effect size and its precision (such as 95% confidence interval)	6–10; Table 2 and Table 3; Figure 2
	17b	For binary outcomes, presentation of both absolute and relative effect sizes is recommended	NA
Ancillary analyses	18	Results of any other analyses performed, including subgroup analyses and adjusted analyses, distinguishing prespecified from exploratory	6–14; Table 2, Table 3 and Table 4; Figure 2 and Figure 3
Harms	19	All important harms or unintended effects in each group (for specific guidance, see CONSORT for harms)	3
**Discussion**			
Limitations	20	Trial limitations, addressing sources of potential bias, imprecision, and, if relevant, multiplicity of analyses	18
Generalisability	21	Generalisability (external validity and applicability) of the trial findings	14–18
Interpretation	22	Interpretation consistent with results, balancing benefits, and harms, and considering other relevant evidence	14–18
**Other information**			
Registration	23	Registration number and name of trial registry	NA
Protocol	24	Where the full trial protocol can be accessed, if available	2–4
Funding	25	Sources of funding and other support (such as supply of drugs), role of funders	19

We strongly recommend reading this statement in conjunction with the CONSORT 2010 Explanation and Elaboration for important clarifications on all the items. If relevant, we also recommend reading CONSORT extensions for cluster randomized trials, noninferiority and equivalence trials, nonpharmacological treatments, herbal interventions, and pragmatic trials. Additional extensions are forthcoming: for those and for up-to-date references relevant to this checklist, see www.consort-statement.org (accessed on 1 May 2022).
